# Use of extended HR-HPV Genotyping in improving the Triage Strategy of 2019 ASCCP recommendations in Women with positive HR-HPV diagnosis and Simultaneous LSIL Cytology Results

**DOI:** 10.7150/jca.55826

**Published:** 2021-05-19

**Authors:** Huifeng Xue, Hangjing Gao, Jinwen Zheng, Yaojia Chen, Jiancui Chen, Diling Pan, Binhua Dong, Pengming Sun

**Affiliations:** 1 Fujian Provincial Cervical Disease Diagnosis and Treatment Health Center, Fujian Maternity and Child Health Hospital, Affiliated Hospital of Fujian Medical University, Fuzhou 350001, Fujian, P.R. China.; 2 Department of Gynecology, Laboratory of Gynecologic Oncology, Fujian Maternity and Child Health Hospital, Affiliated Hospital of Fujian Medical University, Fuzhou 350001, Fujian, P.R. China.; 3 Department of Pathology, Fujian Maternity and Child Health Hospital, Affiliated Hospital of Fujian Medical University, Fuzhou 350001, Fujian, P.R. China.; 4 Fujian Key Laboratory of Women and Children's Critical Diseases Research, Fujian Maternity and Child Health Hospital, Affiliated Hospital of Fujian Medical University, Fuzhou, Fujian 350001, P.R. China.

**Keywords:** human papillomavirus, genotyping, low-grade cervical intraepithelial lesion, cervical intraepithelial neoplasia

## Abstract

**Objective:** According to the 2019 American Society for Colposcopy and Cervical Pathology (ASCCP) recommendations, women with a positive high-risk human papillomavirus (HR-HPV) diagnosis and low-grade cervical intraepithelial lesion (LSIL) cytology result should be referred for further colposcopy examination. However, this strategy results in over-treatment in several cases. In this study, we assessed the performance of extended HR-HPV genotyping in women with a simultaneous positive HR-HPV and LSIL diagnosis with the aim of improving the current triage strategy.

**Methods:** This study was an observational analysis of women from the Fujian Province Cervical Lesion Screening Cohorts (FCLSCs). Women who were HR-HPV-positive and had a cytological examination of LSIL, which were followed up with colposcopy and biopsy, from 2015 to 2018 were included. The study endpoint was defined as the detection of histological cervical intraepithelial neoplasia grade 2 or worse (CIN2+). We combined HR-HPV genotypes according to the prevalence rate in histological CIN2+ and ranked them from high to low to establish HR-HPV genotyping models. Outcomes were assessed with respect to sensitivity, specificity, positive predictive value (PPV), negative predictive value (NPV), and colposcopy referral rate.

**Results:** Overall, 56,788 women undergoing preliminary screening for HR-HPV genotyping were included in this study. Among them, 10,499 women positive for HR-HPV underwent a cytology examination, and 902 women with LSIL cytology diagnosed and subsequent biopsy results were included in the final evaluation. Among these patients, 25.1% (226/902) were found to have CIN2+ in histology. HPV-16, -58, -52, -18, -33, and -31 infections were the most common genotypes, and HPV-16, -18, -58, -33, and -31 (odds ratio [OR] = 5.41, 2.98, 1.38, 1.24, and 1.21, respectively) were associated with the potential for histological CIN2+, from the highest to lowest. In the detection of CIN2+ lesions in HR-HPV-positive LSIL women of different HR-HPV genotyping models, the extended HPV 16/18/31/33/52/58 genotyping model was found to have better efficacy with higher sensitivity (92.9%) and NPV (93.0%), but a significantly lower colposcopy referral rate (74.7%) than the ASCCP-recommended HR-HPV non-genotyping model.

**Conclusion:** For HR-HPV-positive women with LSIL, the HPV 16/18/31/33/52/58 genotyping model can serve as an alternative approach to the ASCCP recommendations, potentially reducing the unnecessary colposcopy referral burden in China.

## Introduction

Cervical cancer is the fourth most frequent cause of death in women 1, with the majority of cases occurring in low- or middle-income countries 2. Fortunately, early detection and preventative measures have been shown to be successful in reducing the progression of premalignant cervical lesions 3. Among all cancer types, cervical lesions are among the most efficiently controlled through appropriate screening measures 4, 5. Currently, effective screening methods include human papillomavirus (HPV) testing, cytology, and colposcopy 6.

Since the publication of the 2015 American Society for Colposcopy and Cervical Pathology (ASCCP) interim guidance, HPV testing has been used as the primary screening strategy for cervical cancer 7. Nevertheless, in most cases, the infections are benign and can be resolved within 2 years without the need for invasive treatment 8, 9. Secondary triage of HPV-positive women is recommended during clinical management, including cytology, which allows deferral of colposcopy for low-risk cases, reducing the long follow-up interval 10.

Low-grade squamous intraepithelial lesion (LSIL), as defined by the Bethesda system, is the second most common cervical abnormality in cytological results 11, 12. According to the most recent 2019 ASCCP risk-based management consensus guidelines for the management of cervical cancer screening abnormalities, HPV-positive women with LSIL have an immediate cervical intraepithelial neoplasia (CIN) 3+ risk of over 4.0%, which necessitates a colposcopy referral 13. However, most LSIL cases do not develop into a clinically significant disease. A previous study demonstrated that the prevalence of high-risk (HR)-HPV could reach 76% in women with LSIL 14, indicating high sensitivity but poor specificity. A large number of negative colposcopy/biopsy results were reported in women with positive HPV and LSIL, which poses a challenge for the limited capacity of colposcopy in most hospital systems and subsequent adverse effects from overtreatment 15, 16. Hence, risk stratification strategies that can better identify underlying or incipient CIN2, CIN3, adenocarcinoma *in situ* (AIS), or cancer (CIN2+) with less resource waste are needed in HPV-positive patients with LSIL. However, most of the related articles published to date have mainly investigated HPV-positive patients with LSIL using the cytology as a primary screening method but not the HPV testing 17-21. Moreover, no study has yet addressed application of the 2019 ASCCP guidelines in such patients.

Extensive evidence indicates that the persistent infection of HPV promotes the progression of cervical cancer 22, 23. However, individual HPV types differ enormously in their relative carcinogenic potential. Thirteen HPV genotypes, including HPV 16, 18, 31, 33, 35, 39, 45, 51, 52, 56, 58, 59, and 66, are categorized as causative agents of cancer; HPV 68 is considered to be probably carcinogenic 23. According to a previously published study, the baseline infection rate of HR-HPV genotypes among women with baseline normal cytology but who subsequently developed CIN3+ during a follow-up period of 11.5 years was as follows: 19.4% HPV-16, 11.7% HPV-18, 13.3% HPV-31, 13.3% HPV-33, 10.7% HPV-52, 9.9% HPV-39, 8.6% HPV-35, 7.9% HPV-58, 7.9% HPV-45, 7.9% HPV-59, 7.7% HPV-51, 7.2% HPV-68, and 6.2% HPV-56 24. This information provides a basis for the selection of HPV genotypes for the appropriate triaging of women with HR-HPV and LSIL cytology.

The purpose of this retrospective study was to assess the potential of extended HR-HPV genotyping in identifying underlying histological CIN2+ in women with a positive HR-HPV and LSIL finding. Furthermore, we aimed to assess the efficacy of different HR-HPV genotyping models to optimize the 2019 ASCCP risk-based management consensus guidelines for these patients to achieve a reduction in the unnecessary use of colposcopy resources.

## Methods

### Patients

**Figure 1:**
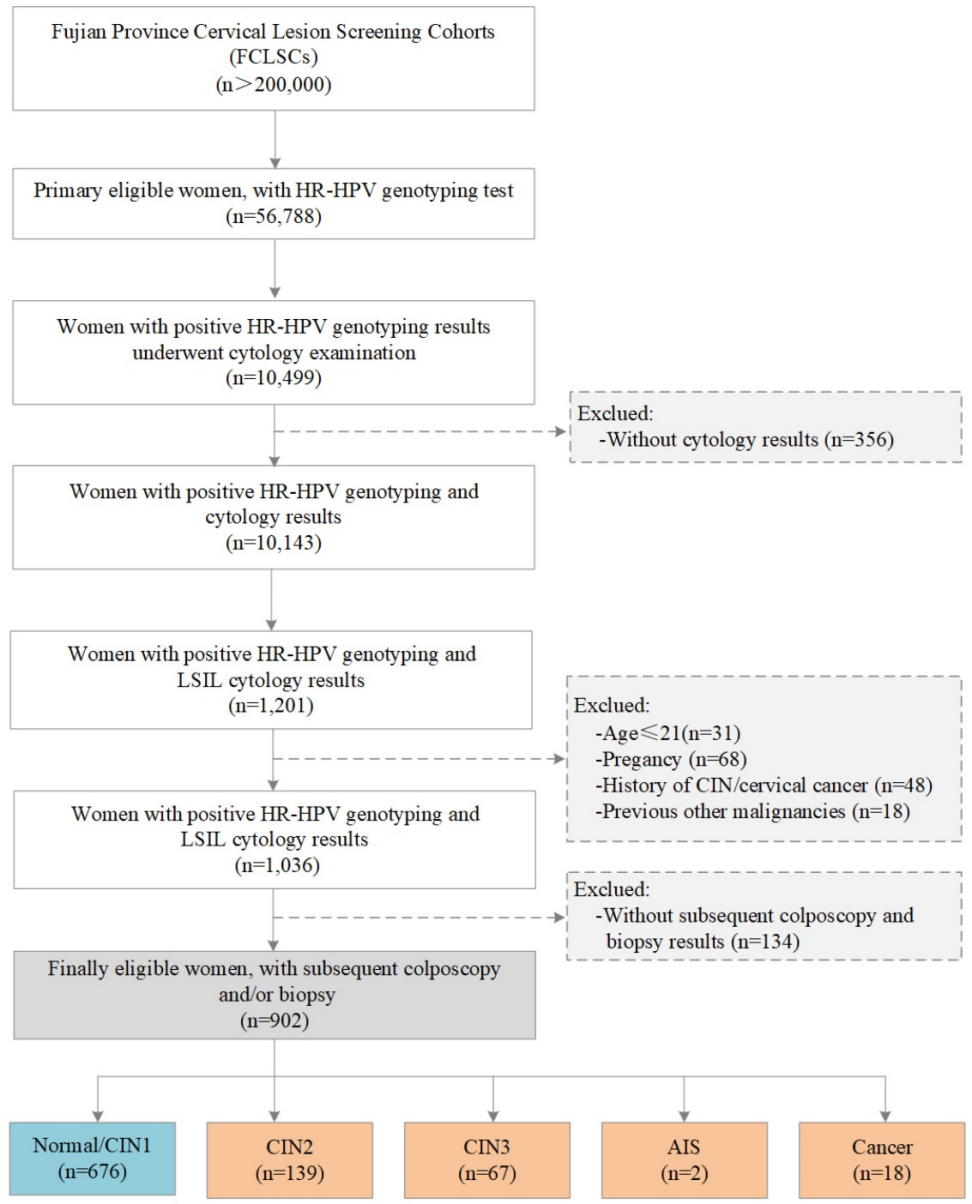
** Flow chart for the selection of patients in this study. Abbreviations:** LSIL, low-grade squamous intraepithelial lesion; HR-HPV, high-risk human papillomavirus; CIN, cervical intraepithelial neoplasia; AIS, adenocarcinoma *in situ*.

The study population was selected from the Fujian Province Cervical Lesion Screening Cohorts (FCLSCs) with more than 200,000 cases, including a provincial-level hospital, nine municipal-level hospitals, and more than 500 community health service centers 25, 26. All participants were required to satisfy the following characteristics: (1) history of sexual activity, (2) willingness to undergo cervical cancer screening with cervical cytology and an HR-HPV genotyping test, (3) no history of severe immunodeficiency disease, and (4) provision of written informed consent. From January 2015 to December 2018, women from the FCLSCs who underwent HR-HPV genotyping were initially included. Women with positive HR-HPV genotyping results subsequently underwent a cytology examination. Women without cytology results were excluded. Subsequent participants were also excluded according to the following criteria: (1) age less than 21 years, (2) pregnancy, (3) history of CIN/cervical cancer, (4) other previous malignancies, and (5) no subsequent colposcopy and biopsy results. The final eligibility criteria included women with an HR-HPV-positive diagnosis with HR-HPV genotyping, LSIL cytology, and subsequent colposcopy and/or biopsy results (Figure 1). The Ethics Committees of the Fujian Maternity and Child Health Hospital approved this study (2014-045).

### Collection of basic information and cervical specimens

Prior to registration, each woman provided informed consent. An experienced doctor conducted a confidential interview with a questionnaire to collect basic information, including the patient's history of medication, cervix-related diseases and treatments, other malignancies, education background, smoking and drinking habits, and reproduction history. All eligible individuals underwent gynecological examinations. Exfoliated cervical cells from the ecto- or endo-cervical canals were obtained with a cytobrush. For HPV genotyping, specimens were stored using a preservation solution in 2 ml vials at -20 °C. For cytology, samples were stored using ThinPrep® PreservCyt® (Hologic, Waltham, MA, USA) in 20 ml vials at 4 °C. Samples were subsequently transferred to the laboratory and cytological site where HR-HPV genotyping and cytology were conducted.

### HR-HPV genotyping test

Polymerase chain reaction-reverse dot blot (PCR-RDB) was used for the analysis of HR-HPV, including 14 HR-HPV genotypes (HPV-16, -18, -31, -33, -35, -39, -45, -51, -52, -56, -58, -59, -66, and -68), in cervical exfoliated cells (Yaneng® Biosciences, ShenZhen, China). All detection procedures were conducted according to the manufacturer's instructions 27.

### Cytology

An auto-image system (Hologic, Inc., San Diego, CA, USA) was used for cervical cytological examinations. All slides prepared for the cytological examinations were analyzed independently by two experienced cytopathologists and were diagnosed according to the Bethesda system 28. If there was divergence in the diagnosis, the cervical samples were re-evaluated to reach a consensus.

### Histology

HR-HPV-infected participants who were also diagnosed with LSIL were subsequently referred for colposcopy and/or biopsy within 10 weeks. Colposcopy results that were deemed normal had no requirement for a biopsy. In contrast, participants with abnormal colposcopy results received an immediate biopsy of visible lesions. The results were interpreted in accordance with the CIN system 29, including normal, CIN1, CIN2, CIN3, AIS, and cancer. If a sample was diagnosed with a primary histology result of CIN2+, the sample was reviewed by another independent histopathologist. Any discrepancy was discussed and resolved by a second histological examination until consensus was reached.

### Statistical analysis

The referral rate was determined by dividing the number of HR-HPV-infected LSIL patients by the overall number of LSIL patients. The mean and standard deviation (SD) of the classified variables were assessed. The values and percentages were calculated. The study endpoint was defined as the detection of histological CIN2+ 10. We combined HR-HPV genotypes according to the prevalence rate in LSIL patients whose biopsy showed CIN2+, ranked from highest to lowest, to establish the HR-HPV genotyping model. According to the guidelines 15, HPV-16- and/or 18-positive patients should be further examined via colposcopy; thus, HPV 16/18 was divided into a separate, priority HR-HPV genotyping model. We assessed the potential of different HR-HPV genotyping models in detecting underlying CIN2+ with respect to their sensitivity, specificity, positive predictive value (PPV), and negative predictive value (NPV), which were then compared to the gold-standard histological diagnosis. The odds ratio (OR) for the incidence of CIN2+ in women with LSIL, accounting for age and HR-HPV positivity, was also calculated. p values < 0.05 (two-sided) were regarded as statistically significant. Statistical analysis was performed using Stata 14 software (Stata Corp., College Station, TX, USA).

## Results

### Baseline characteristics of women with HR-HPV infection and LSIL

A total of 56,788 women with HR-HPV genotyping results who also underwent cervical cytology were recruited for this study. Among these, 10,499 (18.5%) women with positive HR-HPV genotyping results underwent cytology examination, and 356 (3.4%) HR-HPV-positive women without cytology results were excluded. Among the remaining 10,143 (17.9%) HR-HPV-positive women with cytology results, 1,201 (2.1%) women were HR-HPV-positive with both HR-HPV genotyping and LSIL cytology results. After exclusions, 1,036 (1.8%) women were eligible for the study. 134 (12.9%) of whom without colposcopy and/or biopsy results were lost to follow-up and excluded, and the remaining 902 women (87.1%) with colposcopy biopsies were included for final evaluation. The baseline features of the participants are described in Table 1. The average age of the enrolled participants was 38.27 ± 9.86 years (range, 21-74 years). Of the 902 women, 63.2% had received higher education, 97.3% denied a history of smoking, 83.5% denied a history of drinking, and 33.0% had more than two pregnancies.

### Positivity rate of HR-HPV genotypes among HR-HPV-positive participants with LSIL

**Figure 2:**
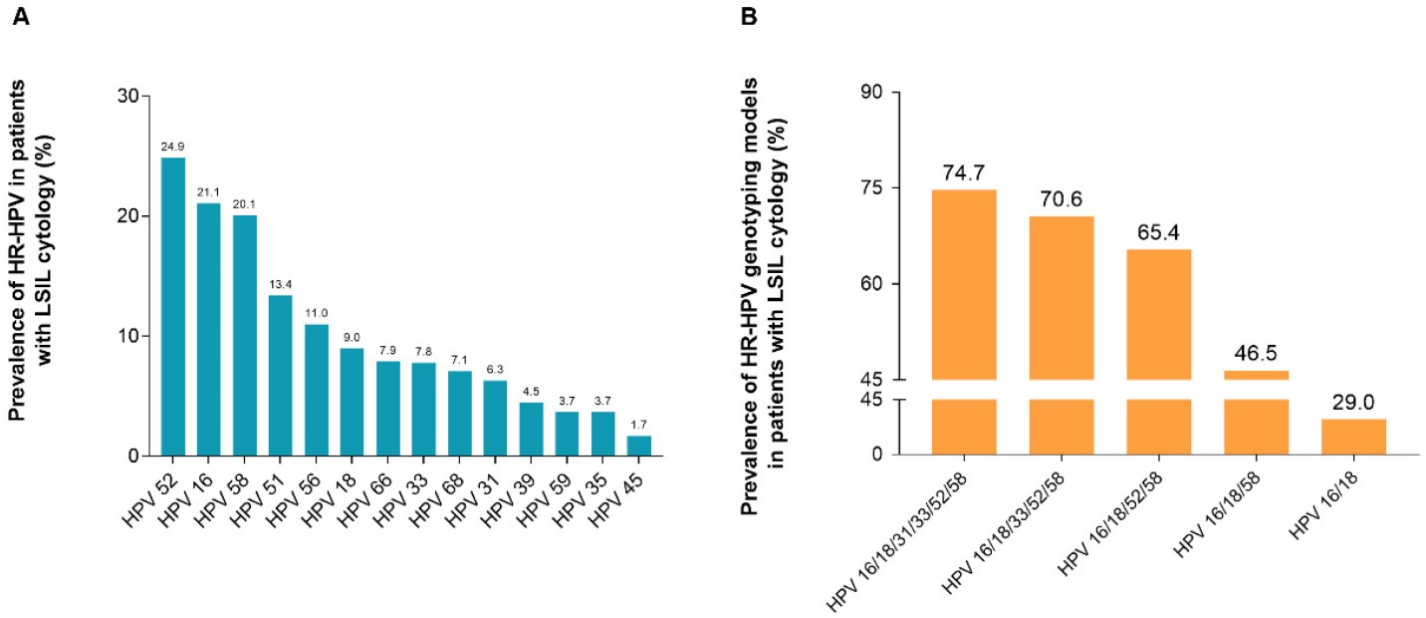
** Positivity rate of HR-HPV genotypes among participants with LSIL cytology.** (A) Prevalence rate of HR-HPV genotypes in 902 women with LSIL. (B) Prevalence rate of HR-HPV genotyping models in 902 women with LSIL. HPV 16/18: women with HPV 16 and/or HPV 18 infection; HPV 16/18/58: women with any infection of HPV-16, -18, -58; HPV 16/18/52/58: women with any infection of HPV-16, -18, -52, -58; HPV 16/18/33/52/58: women with any infection of HPV-16, -18, -33, -52, -58; HPV 16/18/31/33/52/58: women with any infection of HPV-16, -18, -31, -33, -52, -58. **Abbreviations:** HR-HPV, high-risk human papillomavirus; LSIL, low-grade squamous intraepithelial lesion.

Among all HR-HPV-positive participants with LSIL, the most prevalent HR-HPV genotype was HPV-52, accounting for 24.9% (225/902), followed by HPV-16 (190/902, 21.1%), HPV-58 (181/902, 20.1%), HPV-51 (121/902, 13.4%), and HPV-56 (99/902, 11.0%) (Figure 2, Supplement Table S1).

**Figure 3:**
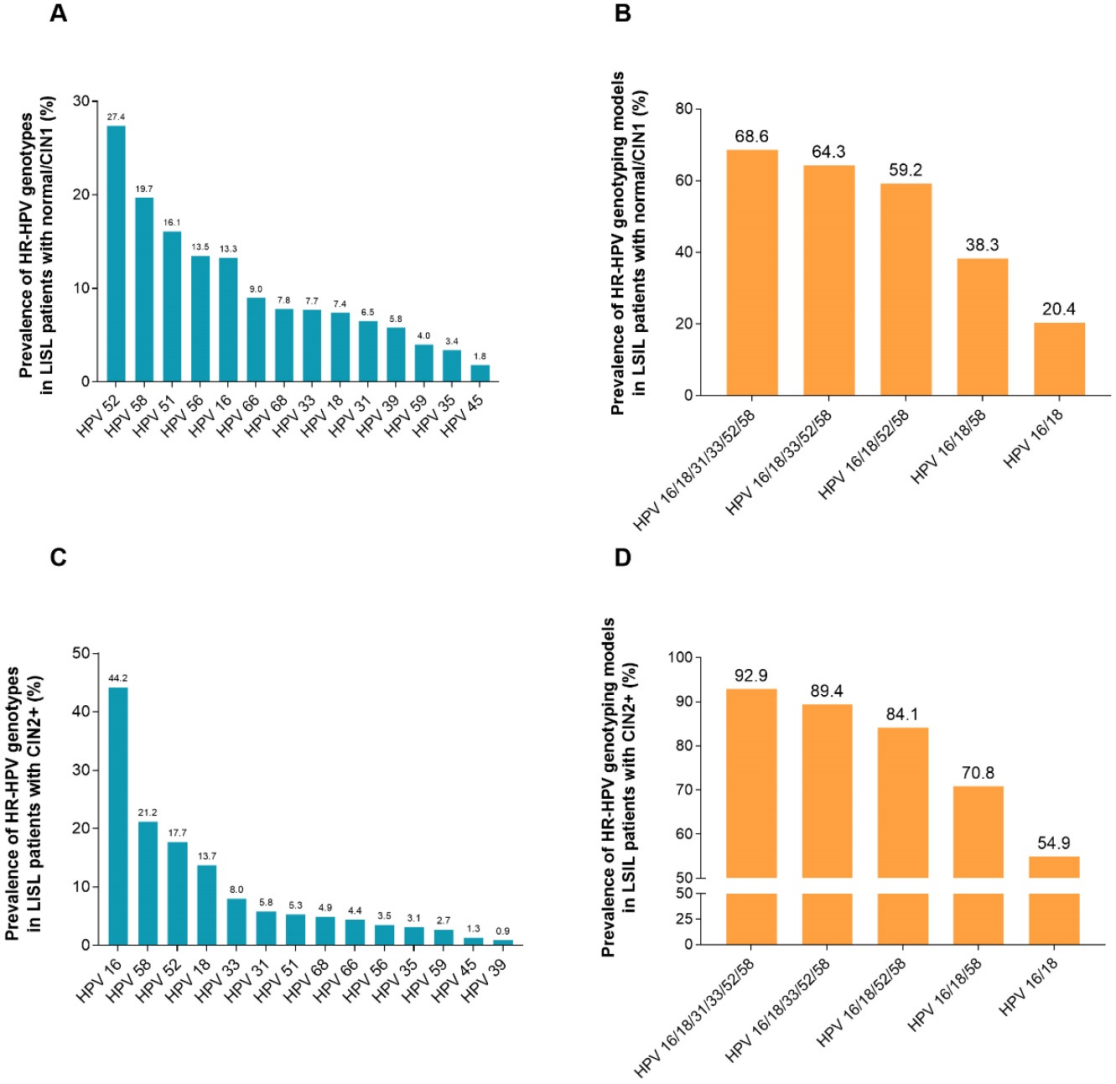
** Prevalence of HR-HPV genotypes based on the histological diagnosis of 902 women with LSIL cytology.** (A) Prevalence of HR-HPV genotypes in LSIL patients with biopsy showing normal/CIN1. (B) Prevalence of HR-HPV genotyping models in LSIL patients with biopsy showing normal/CIN1. HPV 16/18: women with HPV 16 and/or HPV 18 infection; HPV 16/18/58: women with any infection of HPV-16, -18, -58; HPV 16/18/52/58: women with any infection of HPV-16, -18, -52, -58; HPV 16/18/33/52/58: women with any infection of HPV-16, -18, -33, -52, -58; HPV 16/18/31/33/52/58: women with any infection of HPV-16, -18, -31, -33, -52, -58. (C) Prevalence of HR-HPV genotypes in LSIL patients with biopsy showing CIN2+. (D) Prevalence of HR-HPV genotyping models in LSIL patients with biopsy showing CIN2+. **Abbreviations:** HR-HPV, high-risk human papillomavirus; LSIL, low-grade squamous intraepithelial lesion; CIN, cervical intraepithelial neoplasia; CIN2+, cervical intraepithelial neoplasia grade 2 or worse.

Figure 3 (Supplement Table S2) listed the HR-HPV infection rates in HR-HPV-infected women with LSIL and a CIN2+ biopsy. Overall, 25.1% (226/902) women with HR-HPV infection and simultaneous cytology results of LSIL were found to have CIN2+ in histology. The HR-HPV positivity rate in participants with LSIL and a biopsy confirming CIN2+ status was the highest in HPV-16 (44.2%), followed by HPV-58 (21.2%), HPV-52 (17.7%), HPV-18 (13.7%), HPV-33 (8.0%), HPV-31 (5.8%), HPV-51 (5.3%), HPV-68 (4.9%), HPV-66 (4.4%), HPV-56 (3.5%), HPV-59 (3.1%), HPV-35 (3.1%), HPV-45 (1.3%), and HPV-39 (0.9%). The infection rate of HR-HPV increased with the increase in the HR-HPV genotyping models combining more HR-HPV genotypes. Of the participants with LSIL, 92.9% (p < 0.001) can be confirmed as having histological CIN2+ under the HPV genotyping model HPV 16/18/31/33/52/58. However, the rate was only 54.9% (p < 0.001) for the HPV 16/18 genotyping model.

### Odds ratio for histological CIN2+ with different HR-HPV genotypes in participants with LSIL

Table 2 presents the predictive factors for CIN2+ in women with LSIL. After adjusting for age, education level, smoking, drinking, and pregnancies, the infection of HPV-16 was the greatest risk factor for the occurrence of CIN2+ (OR, 5.41; 95% CI, 3.58-8.18; p < 0.001) in women with LSIL. Other HR-HPV genotypes that were correlated with the occurrence of CIN2+ were as follows: HPV-18 (OR, 2.98; 95% CI, 1.69-5.25; p < 0.001), HPV-58 (OR, 1.38; 95% CI, 0.87-2.20; p = 0.168), HPV-33 (OR, 1.24; 95% CI, 0.66-2.31; p = 0.503), and HPV-31 (OR, 1.21; 95% CI, 0.58-2.51; p = 0.611). The remaining HR-HPV genotypes (HPV-35, -39, -45, -51, -52, -56, -59, -66, -68) were reported to have no significant correlation with the occurrence of CIN2+. Moreover, we evaluated the risk of CIN2+ in women with LSIL, according to the HR-HPV genotyping models. The risk assessment of HR-HPV genotyping models was as follows: HPV 16/18 genotyping model (OR, 5.38, 95% CI, 3.83-7.55; p < 0.001), HPV 16/18/58 genotyping model (OR, 4.13, 95% CI, 2.95-5.77; p < 0.001), HPV 16/18/52/58 genotyping model (OR, 3.96, 95% CI, 2.66-5.90; p < 0.001), HPV 16/18/33/52/58 genotyping model (OR, 4.93, 95% CI, 3.11-7.80; p < 0.001), and HPV 16/18/31/33/52/58 genotyping model (OR, 6.19, 95% CI, 3.61-10.63; p < 0.001).

### The accuracy of HR-HPV genotyping models in triaging HR-HPV-positive women with simultaneous LSIL

The sensitivities and NPVs were higher in HR-HPV genotyping models that combined more HR-HPV genotypes, with a decrease in the specificity and PPV. The sensitivity and NPV rates for the extended HPV 16/18/31/33/52/58 genotyping model were 92.9% and 93.0%, respectively, and represented the highest values observed for the detection of underlying CIN2+ pathology, followed by the extended HPV 16/18/33/52/58, HPV 16/18/52/58, HPV 16/18/58, and HPV 16/18 genotyping models. In addition, the colposcopy referral rate of women with the extended HPV 16/18/31/33/52/58 genotyping model was only 74.7% (Table 3).

## Discussion

According to the most current ASCCP risk-based management consensus guidelines, HPV-positive women whose cytology testing reveals LSIL are recommended for further colposcopy because of the greater than 4.0% risk for an immediate CIN3+ 15. However, it should be emphasized that different types of HR-HPV have varying potential with respect to the progression of CIN 23. Triaging all HPV-positive LSIL women to conduct colposcopies will lead to a significant cost burden and overtreatment of patients. Here, we evaluated the correlation between different HR-HPV genotypes and underlying CIN2+ in HPV-positive patients who also had LSIL based on the newly revised 2019 ASCCP risk-based management consensus guidelines, in order to reduce unnecessary colposcopy referrals. Previous reports had focused mainly on HR-HPV or HPV 16/18 30-32, or investigated a population with cytology as a primary screening method. No study has assessed application of the 2019 ASCCP guidelines in women who are HR-HPV-positive with a LSIL cytology until now. Thus, to the best of our knowledge, this is the first investigation on this population with respect to the 2019 ASCCP guidelines. The present results revealed that the extended HPV 16/18/31/33/52/58 genotyping model had better sensitivity and NPV than other HR-HPV genotyping models with respect to CIN2+ status; moreover, the rate of colposcopies was lower than that of transferring all positive HR-HPV for colposcopies.

In our previous study, the most prevalent HR-HPV genotypes identified in the Chinese population were HPV-16, -52, -58, -18, -53, and -33 27. However, we found that HPV-16, -18, -58, -52, -31, and -33, ranked from the highest to lowest, were the most prevalent HR-HPV genotypes in women with LSIL whose biopsy revealed CIN2+. In these patients, the detection rates of HPV-16, -18, -58, -52, -33, and -31 were 44.2%, 13.7%, 21.2%, 17.7%, 8.0%, and 5.8%, respectively. Recent data suggest that the most pathogenic HPV genotypes can be used to identify women who are at high risk of CIN3 33, 34. These findings indicated a higher risk of underlying CIN2+ in patients with simultaneous HPV-16, -18, -31, -33, -52, or -58 infection, which emphasizes the importance of detecting specific HR-HPV genotypes.

We also evaluated the risk of CIN2+ associated with different HR-HPV genotypes. HPV-16 infection was found to have the highest correlation with the detection of CIN2+ in histology. HPV-18, HPV-58, HPV-33, and HPV-31 were also found to be associated with the incidence of histological CIN2+. Combining these HR-HPV genotypes in HR-HPV genotyping models increased the risk of having potential histological CIN2+. According to our results, the HPV 16/18/31/33/52/58 model showed an estimated OR of 6.19 (95% CI, 3.61-10.63) for histological CIN2+.

Women with positive HPV results and LSIL face a significant risk of developing cervical precancerous lesions 35. The sensitivity of HR-HPV testing can reach 100.0%, but at the expense of significant losses in specificity (20.3%) 36. This is important because a test with poor specificity can overestimate the risk of cervical cancer, which can induce anxiety and overtreatment, among other adverse effects 37, 38. Therefore, recommendation of colposcopy and/or biopsy for patients with LSIL who are positive for an HPV infection that falls into the HPV 16/18/31/33/52/58 genotyping model may provide optional management of patient care and hospital resources. All HR-HPV genotyping models were evaluated for the effectiveness of detecting underlying CIN2+ in LSIL patients with respect to sensitivity, specificity, PPV, and NPV. In agreement with previous results, triaging women with LSIL using the extended HPV 16/18/31/33/52/58 genotyping model showed higher sensitivity (92.9% vs. 54.9%) and lower specificity (31.4% vs. 79.6%) than obtained when using the HPV 16/18 genotyping model, leading to reduced risk of misdiagnosis 32. In addition, the extended HPV 16/18/31/33/52/58 genotyping model was also more sensitive to the detection of CIN2+ than any other genotyping model because of the high sensitivity (92.9%). In addition, according to the 2019 ASCCP risk-based management consensus guidelines 13, all HR-HPV-infected women with LSIL had a risk slightly above 4.0% for CIN3+, and were therefore recommended to undergo immediate colposcopy. However, the extended HPV 16/18/31/33/52/58 model indicated a significantly lower referral rate (74.7%), but high sensitivity (92.9%) and NPV (93.0%), which could significantly reduce the burden for health care systems with limited colposcopy capacity but reduced misdiagnosis. Hence, colposcopy referral according to the extended HPV 16/18/31/33/52/58 genotyping model is believed to be a beneficial alternative triaging management approach for Chinese patients.

A potential limitation of this study included the small number of participants with a CIN3+ biopsy. Using histological CIN3+ as the main study outcome can result in an unstable and imprecise sensitivity result. Therefore, our analysis was restricted to CIN2+ as the study endpoint. A second limitation is the enrollment of participants from a single region. In addition, the study lacked large-scale follow-up, which will be conducted in future work.

In conclusion, with a high sensitivity and NPV, but lower referral rates, the extended HPV 16/18/31/33/52/58 genotyping model can provide an alternative to current triaging management of HR-HPV-positive women with LSIL, based on ASCCP guidelines, resulting in a significant reduction in unnecessary referrals for subsequent testing. Future work should focus on the evaluation and validation of this model in large-scale follow-up and different populations.

## Supplementary Material

Supplementary tables.

## Figures and Tables

**Table 1:** Baseline characteristics of 902 women with positive HR-HPV and LSIL

Variates	n (n=902)	Mean (x±s) or prevalence (%)
**Age (years)**		
21-74	902	38.27±9.86
21-30	216	26.59±2.68
31-40	337	35.26±2.87
41-50	248	44.89±2.67
51-65	92	55.83±3.71
>65	9	69.33±2.96
**Education degree**		
Lower education	332	36.8
Higher education	570	63.2
**Drinking**		
Yes	149	16.5
No	753	83.5
**Smoking**		
Yes	24	2.7
No	878	97.3
**Pregnancy**		
≤2	604	67.0
>2	298	33.0

**Abbreviations:** LSIL, low-grade squamous intraepithelial lesion; HR-HPV, high-risk human papillomavirus.

**Table 2:** Odds ratio of histological CIN2+ according to different HR-HPV genotypes among 902 women with LSIL cytology

Variates	OR	OR_adjust_ (95% CI)^a^	p-value
**Age**			
21-30	1 (R)	1 (R)	
31-40	0.77 (0.15-3.86)	0.47 (0.08-2.69)	0.393
41-50	0.89 (0.18-4.36)	0.64 (0.11-3.61)	0.612
51-65	1.93 (0.39-9.47)	1.35 (0.24-7.62)	0.737
>65	1.61 (0.32-8.24)	1.43 (0.24-8.44)	0.697
**HPV16**			
Negative	1(R)	1(R)	
Positive	5.17(3.66-7.29)	5.41(3.58-8.18)	<0.001
**HPV18**			
Negative	1 (R)	1 (R)	
Positive	1.99 (1.24-3.20)	2.98 (1.69-5.25)	<0.001
**HPV31**			
Negative	1 (R)	1 (R)	
Positive	0.88 (0.46-1.66)	1.21 (0.58-2.51)	0.611
**HPV33**			
Negative	1 (R)	1 (R)	
Positive	1.04 (0.759-1.82)	1.24 (0.66-2.31)	0.503
**HPV35**			
Negative	1 (R)	1 (R)	
Positive	0.91 (0.38-2.14)	1.02 (0.40-2.62)	0.961
**HPV39**			
Negative	1 (R)	1 (R)	
Positive	0.18 (0.04-0.73)	0.13 (0.03-0.61)	0.010
**HPV45**			
Negative	1 (R)	1 (R)	
Positive	0.91 (0.26-3.26)	0.80 (0.20-3.19)	0.752
**HPV51**			
Negative	1 (R)	1 (R)	
Positive	0.37 (0.20-0.69)	0.42 (0.22-0.83)	0.012
**HPV52**			
Negative	1 (R)	1 (R)	
Positive	0.57 (0.39-0.84)	0.75 (0.48-1.16)	0.194
**HPV56**			
Negative	1 (R)	1 (R)	
Positive	0.57 (0.29-1.13)	0.26 (0.12-0.59)	0.001
**HPV58**			
Negative	1 (R)	1 (R)	
Positive	1.10 (0.76-1.60)	1.38 (0.87-2.20)	0.168
**HPV59**			
Negative	1 (R)	1 (R)	
Positive	0.66 (0.27-1.61)	0.64 (0.24-1.23)	0.383
**HPV66**			
Negative	1 (R)	1 (R)	
Positive	0.47 (0.24-0.93)	0.58 (0.28-1.23)	0.157
**HPV68**			
Negative	1 (R)	1 (R)	
Positive	0.60 (0.31-1.17)	0.81 (0.39-1.69)	0.579
**HPV16/18^b^**			
Negative	1 (R)	1 (R)	
Positive	4.74 (3.44-6.54)	5.38 (3.83-7.55)	<0.001
**HPV16/18/58^c^**			
Negative	1 (R)	1 (R)	
Positive	3.90 (2.82-5.41)	4.13 (2.95-5.77)	<0.001
**HPV16/18/52/58^d^**			
Negative	1 (R)	1 (R)	
Positive	3.64 (2.47-5.37)	3.96 (2.66-5.90)	<0.001
**HPV16/18/33/52/58^e^**			
Negative	1 (R)	1 (R)	
Positive	4.66 (2.97-7.32)	4.93 (3.11-7.80)	<0.001
**HPV16/18/31/33/52/58^f^**			
Negative	1 (R)	1 (R)	
Positive	6.00 (3.52-10.52)	6.19 (3.61-10.63)	<0.001

**Note:** ^a^: OR values were adjusted for age, education level, smoking, drinking, and number of pregnancy; ^b^: Women with any infection of HPV-16, -18;^ c^: Women with any infection of HPV-16, -18, -58; ^d^: Women with any infection of HPV-16, -18, -52, -58; ^e^: Women with any infection of HPV-16, -18, -33, -52, -58; ^f^: Women with any infection of HPV-16, -18, -31, -33, -52, -58. **Abbreviations:** OR, odds ratio; CI, confidence interval; R; reference; LSIL, low-grade squamous intraepithelial lesion; HR-HPV, high-risk human papillomavirus.

**Table 3:** The accuracy of different HR-HPV genotyping models to triage women with HR-HPV positive and simultaneous LSIL to detect underlying CIN2+

HR-HPV genotyping models	Sensitivity (%)	Specificity (%)	PPV (%)	NPV (%)	Referral rate^a^ (%)
HPV16/18^b^	54.9 (48.1-61.4)	79.6 (76.3-82.5)	47.3 (41.2-53.6)	84.1 (80.9-86.8)	29.0 (262/902)
HPV16/18/58^c^	70.8 (64.3-76.5)	61.7 (57.9-65.3)	38.2 (33.5-43.0)	86.3 (82.9-89.2)	46.5 (419/902)
HPV16/18/52/58^d^	84.0 (78.5-88.5)	40.8 (37.1-44.6)	32.2 (28.5-36.2)	88.5 (84.2-91.7)	65.4 (590/902)
HPV16/18/33/52/58^e^	89.4 (84.4-92.9)	35.7 (32.1-39.4)	31.7 (28.1-35.5)	90.9 (88.7-94.0)	70.6 (637/902)
HPV16/18/31/33/52/58^f^	92.9 (88.5-95.8)	31.4 (27.9-35.0)	31.2 (27.7-34.8)	93.0 (88.6-95.8)	74.7 (674/902)

Note: a: The rate of referred to colposcopy in LSIL women; b: Women with any infection of HPV-16, -18; c: Women with any infection of HPV-16, -18, -58; d: Women with any infection of HPV-16, -18, -52, -58; e: Women with any infection of HPV-16, -18, -33, -52, -58; f: Women with any infection of HPV-16, -18, -31, -33, -52, -58. Abbreviations: HR-HPV, high-risk human papillomavirus; LSIL, low-grade squamous intraepithelial lesion; CIN2+, cervical intraepithelial neoplasia grade 2 or worse; PPV, positive predictive value; NPV, negative predictive value.

## Abbreviations

HR-HPV: high-risk human papillomavirus
LSIL: low-grade squamous intraepithelial lesion
ASCCP: American Society for Colposcopy and Cervical Pathology
AIS: adenocarcinoma *in situ*
CIN: cervical intraepithelial neoplasia
CIN2+: cervical intraepithelial neoplasia grade 2 or worse
PCR-RDB: Polymerase chain reaction reverse dot blot
SD: standard deviation
PPV: positive predictive value
NPV: negative predictive value
OR: odds ratio
CI: confidence interval
