# Supplementary material for: Use of extended HR-HPV Genotyping in improving the Triage Strategy of 2019 ASCCP recommendations in Women with positive HR-HPV diagnosis and Simultaneous LSIL Cytology Results
Source: J Cancer. 2021 May 19;12(14):4332–40. doi: 10.7150/jca.55826 (PMC8176432; doi:10.7150/jca.55826)
Supplement: Supplementary file 1 — Supplementary tables. [file jcav12p4332s1.pdf]

Table S1. Positivity rate of HR-HPV genotypes among 902 women with positive HR-HPV and LSIL cytology

Variates[a]	Positive, n (%)	Negative, n (%)
HR-HPV		
HPV16	190(21.1%)	712(78.9%)
HPV18	81(9.0%)	821(91.0%)
HPV31	57(6.3%)	845(93.7%)
HPV33	70(7.8%)	832(92.2%)
HPV35	30(3.3%)	872(96.7%)
HPV39	41(4.5%)	861(95.5%)
HPV45	15(1.7%)	887(98.3%)
HPV51	121(13.4%)	781(86.6%)
HPV52	225(24.9%)	677(75.1%)
HPV56	99(11.0%)	803(89.0%)
HPV58	181(20.1%)	721(79.9%)
HPV59	33(3.7%)	869(96.3%)
HPV66	71(7.9%)	831(92.1%)
HPV68	64(7.1%)	838(92.9%)
HR-HPV genotyping models		
HPV16/18[b]	262(29.0%)	640(71.0%)
HPV16/18/58[c]	419(46.5%)	483(53.5%)
HPV16/18/52/58[d]	590(65.4%)	312(34.6%)
HPV16/18/33/52/58[e]	637(70.6%)	265(29.4%)
HPV16/18/31/33/52/58[f]	674(74.7%)	228(25.3%)

Note: [a]: Only consider the prevalence of HR-HPV; [b]: Women with any infection of HPV-16, -18; [c]: Women with any infection of HPV-16, -18, -58; [d]: Women with any infection of HPV-16, -18, -52, -58; [e]: Women with any infection of HPV-16, -18, -33, -52, -58; [f]: Women with any infection of HPV-16, -18, -31, -33, -52, -58.

Abbreviations: LSIL, low-grade squamous intraepithelial lesion; HR-HPV, high-risk human papillomavirus

Table S2. Prevalence of HR-HPV genotypes based on the histological diagnosis of 902 women with positive HR-HPV and LSIL cytology

Variates[a]	Histological diagnosis Normal/CIN1 N=676, n(%)	CIN2+ N=226, n(%)	p-value
HR-HPV			
HPV16	90(13.3%)	100(44.2%)	<0.001
HPV18	50(7.4%)	31(13.7%)	0.004
HPV31	44(6.5%)	13(5.8%)	0.686
HPV33	52(7.7%)	18(8.0%)	0.895
HPV35	23(3.4%)	7(3.1%)	0.049
HPV39	39(5.8%)	2(0.9%)	0.001
HPV45	12(1.8 %)	3(1.3%)	0.773
HPV51	109(16.1%)	12(5.3%)	<0.001
HPV52	185(27.4%)	40(17.7%)	0.004
HPV56	91(13.5%)	8(3.5%)	<0.001
HPV58	133(19.7%)	48(21.2%)	0.611
HPV59	27(4.0%)	6(2.7%)	0.353
HPV66	61(9.0%)	10(4.4%)	0.026
HPV68	53(7.8%)	11(4.9%)	0.132
HR-HPV genotyping models			
HPV16/18[b]	138(20.4%)	124(54.9%)	<0.001
HPV16/18/58[c]	259(38.3%)	160(70.8%)	<0.001
HPV16/18/52/58[d]	400(59.2%)	190(84.1%)	<0.001
HPV16/18/33/52/58[e]	435(64.3%)	202(89.4%)	<0.001
HPV16/18/31/33/52/58[f]	464(68.6%)	210(92.9%)	<0.001

Note: [a]: Only consider the prevalence of HR-HPV; [b]: Women with any infection of HPV-16, -18; [c]: Women with any infection of HPV-16, -18, -58; [d]: Women with any infection of HPV-16, -18, -52, -58; [e]: Women with any infection of HPV-16, -18, -33, -52, -58; [f]: Women with any infection of HPV-16, -18, -31, -33, -52, -58.

Abbreviations: HR-HPV, high-risk human papillomavirus; CIN, cervical intraepithelial neoplasia; CIN2+, cervical intraepithelial neoplasia grade 2 or worse; LSIL, low-grade squamous intraepithelial lesion.
